# Testing the ‘microbubble effect’ using the Cavitron technique to measure xylem water extraction curves

**DOI:** 10.1093/aobpla/plw011

**Published:** 2016-02-22

**Authors:** Alexandria L. Pivovaroff, Régis Burlett, Bruno Lavigne, Hervé Cochard, Louis S. Santiago, Sylvain Delzon

**Affiliations:** 1 La Kretz Center for California Conservation Science, University of California Los Angeles, Los Angeles, CA 90095, USA; 2 Université de Bordeaux, UMR BIOGECO, 33405 Talence, France; 3 Department of Botany and Plant Sciences, University of California Riverside, 2150 Batchelor Hall, Riverside, CA 92521, USA; 4 INRA, UMR 547 PIAF, Université Clermont Auvergne, 63100 Clermont-Ferrand, France; 5 INRA, UMR 1202 BIOGECO, 33612 Cestas, France

**Keywords:** Cavitation resistance, embolism, plant hydraulics, vessel length artefact, water relations

## Abstract

Plant resistance to xylem cavitation is a major drought adaptation trait and is essential to characterizing vulnerability to climate change. Cavitation resistance can be determined with vulnerability curves. In the past decade, new techniques have increased the ease and speed at which vulnerability curves are produced. However, these new techniques are also subject to new artefacts, especially as related to long-vesselled species. We tested the reliability of the ‘flow rotor’ centrifuge technique, the so-called Cavitron, and investigated one potential mechanism behind the open vessel artefact in centrifuge-based vulnerability curves: the microbubble effect. The microbubble effect hypothesizes that microbubbles introduced to open vessels, either through sample flushing or injection of solution, travel by buoyancy or mass flow towards the axis of rotation where they artefactually nucleate cavitation. To test the microbubble effect, we constructed vulnerability curves using three different rotor sizes for five species with varying maximum vessel length, as well as water extraction curves that are constructed without injection of solution into the rotor. We found that the Cavitron technique is robust to measure resistance to cavitation in tracheid-bearing and short-vesselled species, but not for long-vesselled ones. Moreover, our results support the microbubble effect hypothesis as the major cause for the open vessel artefact in long-vesselled species.

## Abstract (precis)

Xylem vulnerability to cavitation is an important trait in characterizing woody species' drought tolerance. However, artifacts arise for long-vesselled species when using the *in situ* flow centrifuge method, also known as the Cavitron. We tested the role of microbubbles as a potential mechanism for this bias by constructing vulnerability and xylem water extraction curves for species with different maximum vessel lengths in different rotor sizes. Our results show a major difference in xylem vulnerability to cavitation for long-vesselled species between methods and support the microbubble effect hypothesis.

## Introduction

The ability of plants to resist xylem cavitation and endure periods of water deficit is critical for survival under changing climate conditions (Brodribb and Cochard 2009; Brodribb *et al*. 2010; Choat *et al*. 2012; Urli *et al*. 2013) that may include more extreme events, such as exceptional drought (IPCC 2014). Extreme drought may push species beyond critical thresholds, leading to dieback. Regional vegetation mortality events in response to global climate change-type drought have already been reported on every wooded continent (Allen *et al*. 2010), which have implications for carbon and water cycling and biodiversity. Hence, understanding how plants cope with drought is vital for understanding which species or regions may be most vulnerable. Vulnerability to xylem cavitation is a linchpin trait in characterizing overall plant drought adaptation (Alder *et al*. 1997). Maintaining adequate water transport within the plant is essential for nearly all major functions (Santiago *et al*. 2004; Brodribb 2009). However, during periods of water deficit, water potentials within the xylem can drop to critical thresholds leading to cavitation, or air-filled spaces that disrupt water transport within the xylem conduits (Sperry and Tyree 1988; Brodribb and Cochard 2009; Brodribb *et al*. 2010; Urli *et al*. 2013). Different species have different critical water potential thresholds beyond which cavitation occurs. Typically, the water potential at which 50 % of hydraulic conductivity is lost (*P*_50_) is used to compare cavitation resistance between species.

**Figure 1.**
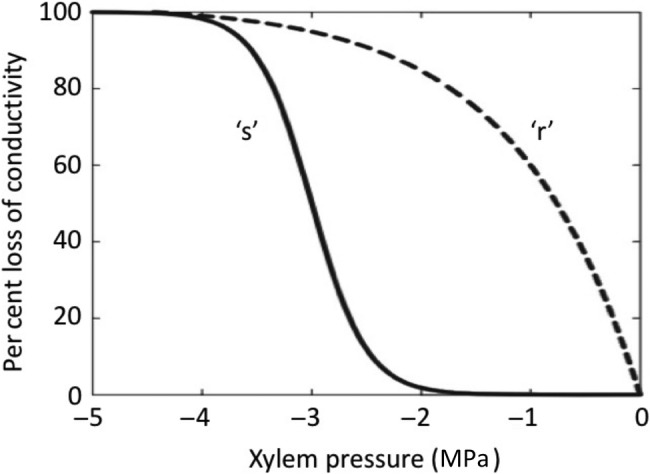
Representative vulnerability curves showing the change in PLC versus xylem pressure for ‘s’-shaped curves (solid line) and ‘r’-shaped curves (dashed line). ‘r’-shaped vulnerability curves are significantly more vulnerable to cavitation than ‘s’-shaped curves. Redrawn from Cochard *et al*. (2010*a*).

The value of *P*_50_ is calculated from a vulnerability curve (Fig. 1), which plots the change in per cent loss of conductivity (PLC) as a function of xylem pressure. There are three principal techniques for inducing cavitation in samples, including bench dehydration, air injection and centrifugation (Cochard *et al*. 2013). Centrifugation-generated vulnerability curves can be constructed using a ‘static rotor’ or a ‘flow rotor’ known as the Cavitron. In the Cavitron, samples are spun at known speeds to induce a negative pressure, but instead of removing the sample from the rotor to measure conductivity in-between pressure steps as is done with the static rotor (Alder *et al*. 1997; Torres-Ruiz *et al*. 2014), water is injected into the Cavitron to measure flow through the sample while it is spinning. Centrifuge-generated vulnerability curves can be constructed much more quickly and with less plant material than the original ‘gold standard’ bench dehydration method. Additionally, by eliminating the need to remove and re-mount samples in between each pressure step as done with the static rotor, the Cavitron has the major advantages of speed and the ease at which vulnerability curves can be generated, allowing high throughput. Direct comparison of the bench dehydration, static rotor and Cavitron methods shows that they produce similar results across a wide range of xylem functional types; the only exception is for long-vesselled species (Cochard *et al*. 2005; Li *et al*. 2008; Martin-StPaul *et al*. 2014).

Recent tests of the centrifuge technique against other independent techniques, including non-invasive methods, have shown that vulnerability curves generated with centrifugation suffer from artefacts when applied to long-vesselled species (Choat *et al.* 2010; Cochard *et al*. 2010*a*; McElrone *et al*. 2012; Delzon and Cochard 2014; Torres-Ruiz *et al*. 2014). At the heart of this artefact is the issue of open vessels, which occur in stem samples where the maximum vessel length exceeds the sample size, leading to xylem vessels that are unobstructed by vessel end-walls or that only have a single vessel end-wall (open from sample end to the middle of the segment). Too many open vessels are thought to lead to anomalous ‘r’-shaped vulnerability curves that over-estimate vulnerability to cavitation (Choat *et al*. 2010; Martin-StPaul *et al*. 2014). A review of all available vulnerability data supports the conclusion that all centrifuge methods are prone to artefact when applied to long-vesselled species (Cochard *et al*. 2013).

Why would open vessels lead to anomalous ‘r’-shaped vulnerability curves? One hypothesized mechanism may be the role of microbubbles (Cochard *et al*. 2005; Sperry *et al*. 2012; Wang *et al*. 2014*a*), which we term the ‘microbubble effect’. The microbubble effect may occur when any solution not previously filtered by an intervessel pit membrane is potentially contaminated with either microbubbles or dust motes that serve as embolism nuclei (Sperry *et al*. 2012; Rockwell *et al*. 2014; Wang *et al*. 2014*a*). Two possible contaminant sources are (i) the solution in the centrifuge reservoirs or (ii) the solution used to measure flow or flush samples (Sperry *et al*. 2012; Rockwell *et al*. 2014). Microbubbles can move in open vessels by buoyancy or mass flow towards the sample centre or axis of rotation in a spinning sample until either the pressure reaches a critical threshold, causing them to expand and form artefactual emboli, or their movement is stopped by an end-wall (Sperry *et al*. 2012; Rockwell *et al*. 2014; Zhang and Holbrook 2014). Species with short vessel lengths would have intact vessels with end-walls, only allowing solution to travel between vessels via pit membranes that can filter solution and impede the movement of microbubbles. In species with long vessels that would be cut open during sample preparation, the lack of end-walls (or having only a single vessel end-wall) means solution with microbubbles or contaminants can enter these vessels without being filtered, magnifying the microbubble effect. This was tested in one experiment by Wang *et al.* (2014*a*), who spun stems of a long-vesselled species, *Robinia pseudoacacia*, at tensions too low to induce ‘real’ cavitation (0.031 MPa) for 4 h and observed a decline in stem conductivity over time as artefactual cavitation occurred. In theory, the microbubble effect would lead to a similar artefact whether the static rotor or Cavitron method is used.

To test the microbubble effect, we constructed vulnerability curves in three different rotor sizes, as well as native and vacuum degassed xylem water extraction curves for five species with varying vessel lengths. In comparing vulnerability curves among three rotor sizes, we hypothesized that when the maximum vessel length exceeded the rotor size, curves would become ‘r’ shaped, altering *P*_50_. Native xylem water extraction curves are not subject to the microbubble effect as no solution is introduced and there is no flow in the sample. Hence, in a second test, we compared vulnerability curves to native extraction curves to determine when artefactual cavitation is occurring, hypothesizing that for short-vesselled species, *P*_50_ obtained from the two methods would agree while there would be a difference for long-vesselled species. Finally, we compared native versus vacuum degassed xylem water extraction curves to test the effects of flushing on curve shape and different water storage phases, hypothesizing that flushing samples introduces microbubbles and magnifies their effect, altering water storage phases and water extraction curve shape. Accurately constructing vulnerability curves is essential to correctly characterizing xylem vulnerability to cavitation, and understanding the mechanisms underlying these potential artefacts can potentially lead to technique improvements and artefact solutions.

## Methods

### Study species

Experiments were performed on five different species with varying conduit lengths, from tracheid (a few millimetres) to vessels >1 m in length, harvested on the campus of University of Bordeaux, Talence, France. Study species in order of increasing vessel length included *Pinus pinaster* Aiton, *Populus nigra* L., *Fagus sylvatica* L., *Prunus cerasifera* Ehrh. and *Eucalyptus* sp.

### Maximum vessel length

Maximum vessel length was determined using the air infiltration technique (Zimmermann and Jeje 1981; Ewers and Fisher 1989) by collecting long stem samples (>1 m) and injecting compressed air (0.1 MPa) into the basal end with the distal end submerged in water. The distal end was cut under water in 5 cm increments until air bubbles were observed, indicating open vessels. Hence, the remaining uncut shoot length constituted the maximum vessel length.

### Xylem vulnerability curves

Vulnerability curves were constructed using the Cavitron technique (Cochard 2002; Cochard *et al*. 2005) using a temperature-controlled centrifuge (Sorvall RC5+, Thermo, USA) equipped with a camera (Scout Sc640gm, Basler, Germany). A custom software (Cavisoft v.4.0, Université de Bordeaux) was used for parameter control and data acquisition (detailed description of this set-up can be found in Wang *et al*. 2014*b*; R. Burlett, H. Inchauspé, J. M. Torres-Ruiz, R. Souchal, H. Cochard and S. Delzon, in prep.). Samples (∼1 m in length) were harvested and the leaves immediately removed before being brought back to the laboratory. Samples were immersed in water and cut to the corresponding length depending on which diameter rotor was to be used, with the sample ends cleanly cut with a fresh razor blade. Rotor diameters and therefore stem sample lengths included 14, 27 and 42 cm. Around 3 cm of bark were removed from each end of the stem samples to fit inside the cuvettes or reservoirs. Samples were placed in the rotor and spun at low speeds producing only moderately negative pressure to first measure the initial stem conductivity (*K*_max_) by injecting a 10 mM KCl and 1 mM CaCl_2_ solution into the Cavitron that flowed through the stem. The rotational speed was then increased in a stepwise manner to measure PLC as:

$$\text{PLC}=100\times \left(1-\frac{K}{K_{\max}}\right)$$

where *K* is hydraulic conductivity. Curves were conducted until >90 % PLC was reached or when the maximum rotational velocity for the Cavitron was achieved, whichever came first. For safety reasons, the minimum xylem pressure in the 14 cm rotor was −3.2 MPa. Vulnerability curves were constructed by plotting PLC versus xylem pressure and fitting a sigmoid function using SAS ver. 9.2 to calculate *P*_12_, *P*_50_ and *P*_88_, the xylem pressures at which 12, 50 and 88 % of hydraulic conductivity is lost, respectively.

### Xylem water extraction curves

For xylem water extraction curves, sample collection and preparation was the same as for vulnerability curves (see above), but differed in that all bark was completely removed from the samples to lower branch symplasmic water content. In addition, we had two treatments for our water extraction curves: native and vacuum degassed. Native samples were not flushed and contained only native sap. Vacuum degassed stem samples were immersed in 10 mM KCl and 1 mM CaCl_2_ solution (the same solution used to measure flow in vulnerability curves) and placed under a relative vacuum of −700 mbar with a pump (N035, KNF, Germany) for >2 h or until all native emboli were removed. Samples were then placed in the 27 cm rotor with intact cuvette reservoirs, along with a small amount of solution. Samples were initially spun at −0.1 MPa to visualize the menisci, after which the speed was increased in a stepwise fashion. At each pressure step, meniscus position was measured with a resolution of 15 μm pixel^−1^ using a digital camera (Scout Sc640gm, Basler), fitted with a C-mount lens (HF16 HA-1B, Fujinon, Japan). Data acquisition and parameter control were performed with a custom software (Cavisoft v.4.0, Université de Bordeaux). Stems were spun at each pressure step until the menisci no longer moved, meaning no water was being released and that equilibrium was achieved (typically ∼2 min). This was repeated until water release became negligible or until the menisci became completely separated, indicating complete sample cavitation. Water extraction curves were calculated by first removing points between 0 and −0.8 MPa to exclude elastic water storage (Tyree and Yang 1990), then fitting a sigmoid function to the remaining points using SAS ver. 9.2 to calculate $P_{50}^{\prime },$ the water potential at which 50 % of xylem water was released. Additional native water extraction curves for *Pinus* and *Prunus* were measured using the 14 cm rotor to test whether water extraction curve shape may shift between ‘s’ and ‘r’ shaped within a species. For safety reasons, the minimum xylem pressure in the 14 cm rotor was −3.2 MPa; these curves were not run to full sample cavitation and water release in order to calculate per cent water extracted or $P_{50}^{\prime }.$ However, the difference in curve shape, as well as differences in water storage phases, can be determined by plotting the ‘raw’ curves (position of the meniscus at each pressure step).

### Vessel anatomy

To measure vessel lumen diameter, four to five cross-sections were cut from each stem used for water extraction curves using a sliding microtome (GSL1 Microtome, Schenkung Dapples, Switzerland). Cross-sections were stained with safranin (1 %), fixed on microscope slides and observed with a light microscope (DM2500, Leica, Germany). Photos of each section were taken with a digital camera (DFC290, Leica) interfaced with computer software (Leica QWin v.3). Images were analysed with ImageJ (v.1.49h).

### Statistical analyses

Vulnerability and extraction curves were constructed by plotting xylem pressure versus PLC or per cent water extracted, respectively. We then fit a sigmoid function using SAS ver. 9.2 to calculate *P*_50_ and $P_{50}^{\prime },$ respectively. Significant differences for *P*_50_ and $P_{50}^{\prime }$ between methods were assessed for each species using a one-way analysis of variance and Tukey Honest Significant Difference *post hoc* test in RStudio ver. 0.99.485 (R Development Core Team 2015). To compare the results obtained from vulnerability curves and extraction curves, we plotted *P*_50_ and $P_{50}^{\prime }$ obtained using the 27 cm diameter rotor for each species and fit a linear regression, excluding *Eucalyptus*.

## Results

### Comparing vulnerability curves among three rotor sizes

**Figure 2.**
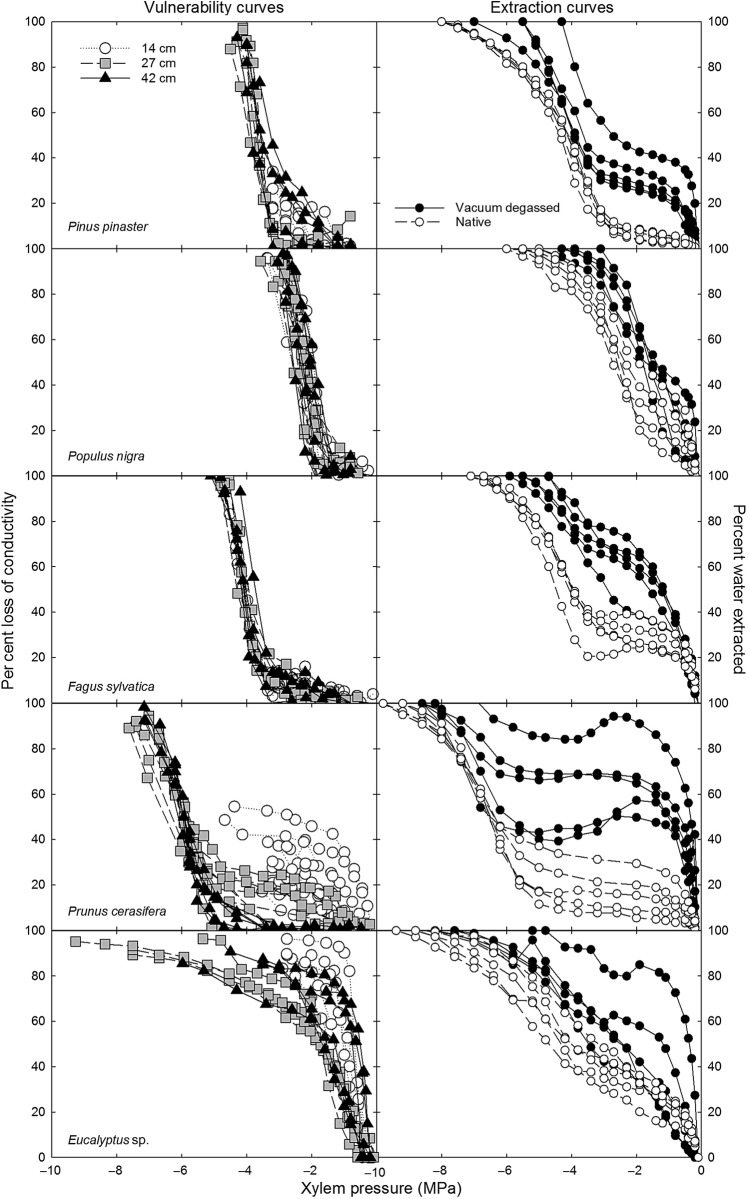
Vulnerability curves (left) and xylem water extraction curves (right) obtained with the *in situ* flow centrifuge technique (Cavitron). Vulnerability curves were constructed using the 14 cm diameter (open circles), 27 cm diameter (grey squares) and 42 cm diameter (black triangles) rotors in the Cavitron for *Pinus pinaster* (14 cm, *n* = 5; 27 cm, *n* = 5; 42 cm, *n* = 5), *Populus nigra* (14 cm, *n* = 5; 27 cm, *n* = 9; 42 cm, *n* = 6), *F. sylvatica* (14 cm, *n* = 5; 27 cm, *n* = 6; 42 cm, *n* = 5), *Prunus cerasifera* (14 cm, *n* = 10; 27 cm, *n* = 9; 42 cm, *n* = 6) and *Eucalyptus* sp. (14 cm, *n* = 6; 27 cm, *n* = 4; 42 cm, *n* = 6). Xylem water extraction curves were constructed using the 27 cm diameter rotor for native (open circles) and vacuum degassed (filled circles) samples of *Pinus pinaster*, *Populus nigra*, *F. sylvatica*, *Prunus cerasifera* and *Eucalyptus* sp., replicated five times for each species and each treatment.

Maximum xylem conduit length varied between the five study species, from tracheids that are only 0.15 cm long for the coniferous *Pinus* to vessels 75 cm long for *Eucalyptus* (*Pinus*< *Populus*< *Fagus*< *Prunus*< *Eucalyptus*; Table 1). *Pinus*, *Populus* and *Fagus* all had ‘s’-shaped curves in all three rotor sizes, while *Eucalyptus*, which has extremely long vessels, had ‘r’-shaped curves in all three rotors (Fig. 2). *Prunus*, which has an intermediate vessel length, displayed a shift in vulnerability curve shape, from an ‘r’-shaped curve in the 14 cm diameter rotor, where the maximum vessel length exceeded the sample length, to an ‘s’-shaped curve in the 42 cm diameter rotor, where the maximum vessel length was less than the sample length.

**Table 1.** Range of maximum vessel lengths and replication, and mean vessel diameters ± SE and replication for five study species.

Species	Maximum vessel length (*n*), cm	Mean vessel diameter ± SE (*n*), μm
*Pinus pinaster*	0.15 (5)	15.8 ± 0.188 (5)
*Populus nigra*	20 (10)	40.6 ± 0.670 (5)
*Fagus sylvatica*	25 (5)	28.4 ± 0.430 (5)
*Prunus cerasifera*	40 (5)	28.9 ± 0.377 (5)
*Eucalyptus* sp.	75 (5)	62.7 ± 0.687 (5)

Values of *P*_50_ measured with the 42 cm rotor varied between species, from −2.22 MPa for *Populus* to −5.95 MPa for *Prunus* (Table 2). For tracheid-bearing and short-vesselled species that did not display any shift in vulnerability curve shape between rotor sizes, there was also no change in the *P*_50_ between rotor sizes, as seen with *Pinus*, *Populus* and *Fagus*. However, when species had an intermediate vessel length and did display a shift in vulnerability curve shape between rotor sizes, they also displayed a change in *P*_50_ (Table 2). This is exemplified by *Prunus*, which had an ‘r’-shaped vulnerability curve and a more vulnerable *P*_12_ of −1.05 MPa and *P*_50_ of −4.31 MPa in the 14 cm diameter rotor, but when a larger rotor was used, the curves became ‘s’ shaped and the *P*_12_ and *P*_50_ more resistant to −4.32 MPa and −5.90 (*P* < 0.001) in the 27 cm rotor, respectively, and to −5.16 and −5.95 MPa (*P* < 0.001) in the 42 cm rotor, respectively (Table 2) **[see Supporting Information—Table S1]**. *Eucalyptus*, a long-vesselled species, always had ‘r’-shaped vulnerability curves and maintained a highly vulnerable *P*_50_ and no difference in *P*_12_ between rotor sizes (Table 2) **[see Supporting Information—Table S1]**.

**Table 2.** The mean water potential at which 50 % of hydraulic conductivity is lost (*P*_50_), with standard error and replicates (*n*), determined from vulnerability curves using the 14, 27 and 42 cm diameter rotors, and the mean water potential at which 50 % of xylem water of released determined from native xylem water extraction curves using the 27 cm diameter rotor, with standard error and replicates (*n*), for five study species. Differences in *P*_50_ among rotor sizes within each species are indicated by dissimilar letters (*P* < 0.05). The value of *P*_50_ measured in the 14 cm diameter rotor for *Pinus* and *Fagus* could not be determined as curves were not run to completion due to the maximum rotational velocity of the rotor.

Species	Vulnerability curves			Extraction curves
	14 cm rotor	27 cm rotor	42 cm rotor	27 cm rotor
*Pinus pinaster*	–	−3.70 ± 0.06 (5)a	−3.50 ± 0.12 (5)a	−4.41 ± 0.05 (5)b
*Populus nigra*	−2.19 ± 0.10 (5)a	−2.32 ± 0.05 (9)a	−2.22 ± 0.10 (6)a	−2.84 ± 0.09 (5)b
*Fagus sylvatica*	–	−4.05 ± 0.05 (6)a	−4.01 ± 0.08 (5)a	−4.54 ± 0.08 (5)b
*Prunus cerasifera*	−4.31 ± 0.28 (10)a	−5.90 ± 0.06 (9)b	−5.95 ± 0.04 (6)b	−6.75 ± 0.05 (5)b
*Eucalyptus* sp.	−1.10 ± 0.13 (6)a	−2.07 ± 0.09 (4)b	−1.34 ± 0.24 (6)a,b	−4.76 ± 0.22 (5)c

### Comparing native water extraction curves with vulnerability curves

**Figure 3.**
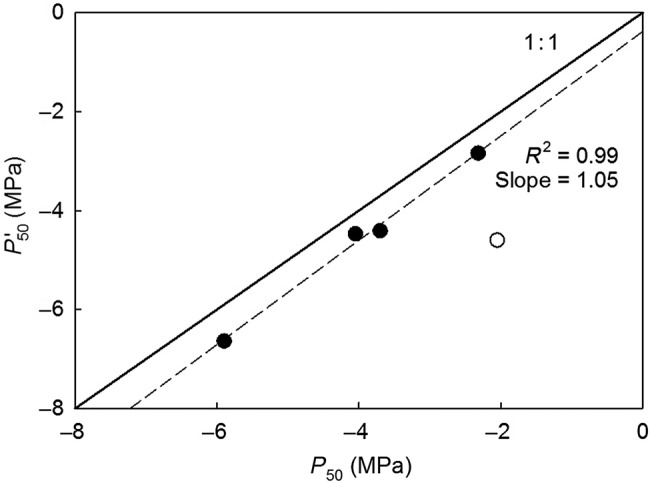
The water potential at which 50 % of hydraulic conductivity is lost (*P*_50_) as calculated from vulnerability curves versus the water potential at which 50 % of xylem water was released ($P_{50}^{\prime }$) as calculated from native water extraction curves, both constructed using the 27 cm diameter rotor in the Cavitron (dashed line). The solid line represents the 1 : 1 line. *Eucalyptus* (open circle) is not included in the linear regression.

Native water extraction curves corrected for elastic water storage tended to be ‘s’ shaped and displayed different phases of water storage, as proposed by Tyree and Yang (1990). $P_{50}^{\prime }$ varied between −2.84 MPa for *Populus* and −6.64 MPa for *Prunus* (Table 2). In comparing *P*_50_ and $P_{50}^{\prime }$ obtained with the 27 cm diameter rotor, they were tightly correlated (*R*^2^ = 0.99; Fig. 3) when long-vesselled *Eucalyptus* was excluded as an outlier. However, $P_{50}^{\prime }$ was always more negative than *P*_50_, except for *Prunus* that had no significant difference between $P_{50}^{\prime }$ and *P*_50_ in the 27 and 42 cm rotors. *Pinus* shifted water release phases around −3 MPa, which is just prior to its *P*_50_ of −3.70 MPa. *Populus* shifted to cavitation water release around −1.5 MPa, which is just prior to its *P*_50_ of −2.31 MPa. *Fagus* shifted around −3.0 MPa, just prior to its −4.05 MPa *P*_50_. According to its native water extraction curves, *Prunus* did not experience significant cavitation until −5 MPa, which is just prior to the *P*_50_ measured using the 27 and 42 cm diameter rotors of −5.90 and −5.95 MPa, respectively, but is after the 14 cm *P*_50_ of −4.31 MPa. For *Eucalyptus*, it was difficult to identify the shift to cavitation water release. However, the $P_{50}^{\prime }$ was approximately twice as negative as the *P*_50_ obtained with the 27 cm diameter rotor.

### Comparing native versus vacuum degassed water extraction curves

**Figure 4.**
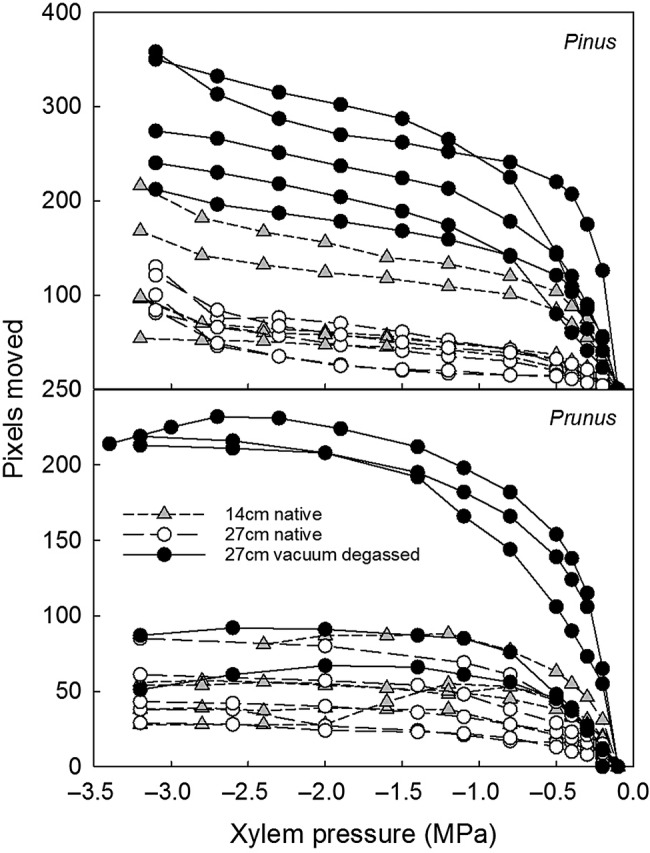
Xylem water extraction curves plotted as xylem pressure versus number of pixels the menisci moved (a proxy for volume of water released) for *Pinus* and *Prunus* for 27 cm long vacuum degassed samples, 27 cm long native samples and 14 cm long native samples. As the maximum rotational velocity of the 14 cm diameter rotor is 10 000 r.p.m., the maximum pressure was −3.2 MPa. Hence, water extraction curves constructed with the 14 cm rotor could not be run to full sample cavitation and water release, and per cent water extracted could not be calculated.

Overall, water extraction curves were highly repeatable (Fig. 4). However, there was greater variability between replicates in vacuum degassed water extraction curves than native curves. There were shifts in the range of water storage phases between the native and vacuum degassed treatments, with vacuum degassed water extraction curves releasing much more water during the initial phase than the native curves. Manipulating the sample length for native water extraction curves for *Pinus* and *Prunus* led to no differences in water extraction curve shape or shift in the initial water storage phase (Fig. 4); only the vacuum degassed treatment showed a curve shift.

## Discussion

In testing the magnitude of the open vessel artefact, we did not find any change in the shape of vulnerability curves between different rotor sizes for tracheid-bearing, short-vesselled and very long-vesselled species. Tracheid-bearing and short-vesselled species, including *Pinus*, *Populus* and *Fagus*, always produced ‘s’-shaped vulnerability and had no change in *P*_50_ no matter the rotor diameter. This indicates that the Cavitron technique is robust to measure resistance to cavitation in these tracheid-bearing and short-vesselled species. For *Eucalyptus*, the case is opposite as it always produced ‘r’-shaped curves as it also always had a maximum vessel length longer than the available rotor sizes. In addition, *Eucalyptus* xylem water extraction curves showed that the *P*_50_ obtained from vulnerability curves did not occur during the cavitation water release phase, providing evidence that the ‘r’-shaped vulnerability curve and resulting *P*_50_ is anomalous. Only with *Prunus*, which has an intermediate vessel length, did we find a change in the shape of the vulnerability curve between rotor sizes. In the 42 cm diameter rotor, *Prunus* would have few or no open vessels, where it also produced an ‘s’-shaped vulnerability curve and a more resistant *P*_50_. In the 14 cm diameter rotor, where it displayed an ‘r’-shaped vulnerability curve and more vulnerable *P*_50_, *Prunus* would be subject to the open vessel artefact. Overall, these results support the findings of Cochard *et al*. (2010*a*), who also showed an increased incidence of ‘r’-shaped curves as maximum vessel size exceeds rotor diameter.

Xylem water extraction curves displayed different phases of water storage, as proposed by Tyree and Yang (1990), including the initial phase of water released from living cells and intercellular spaces and the later phase of water released via cavitation (Vergeynst *et al*. 2014). Furthermore, there was no shift in water extraction curve shape within a species between rotor sizes. For example, while there was a shift in the vulnerability curve shape from ‘s’ in the 27 cm rotor to ‘r’ in the 14 cm rotor for *Prunus*, there was no shift in water extraction curve shape between these two rotor sizes for *Prunus*. These results indicate that water extraction curves are not subject to an open vessel bias like vulnerability curves are. In addition, our tight correspondence between *P*_50_ and $P_{50}^{\prime },$ excluding *Eucalyptus* from the regression as an outlier, is consistent with previous studies (Beikircher *et al*. 2010; Cochard *et al*. 2010*b*). However, within a species, $P_{50}^{\prime }$ was more resistant than *P*_50_, possibly because the extraction method continues to remove water from the stem even after 100 % of the xylem is embolized in the central part of the sample, resulting in complete loss of conductivity in vulnerability curves. Additionally, calculating $P_{50}^{\prime }$ using the volume of water extracted does not take into account xylem redundancy. The discrepancy between *P*_50_ and $P_{50}^{\prime }$ provides evidence that water extraction curves are not a new way to determine vulnerability to cavitation.

Extraction curves measure the water released by the sample at each pressure step, also known as capacitance (Meinzer *et al*. 2003; Vergeynst *et al*. 2014). Water stored in living cells and intercellular spaces is first released at less negative pressures, followed by water released via cavitation at more negative tensions. As the later stage of water release is due to cavitation (Tyree and Yang 1990; Vergeynst *et al*. 2014) and this methodology is not subject to the microbubble effect because the sample is never perfused or flushed with solution, it is possible to compare the pressure at which water released due to cavitation occurs in water extraction curves with vulnerability curves to determine whether the origin of the observed cavitation represents *in planta* processes or an artefact, especially for ‘r’-shaped vulnerability curves. For *Pinus*, *Populus* and *Fagus*, the shift from the initial water release phase to the later cavitation release phase in the water extraction curves occurs prior to the *P*_50_ from the vulnerability curve, indicating the loss of conductance observed in the vulnerability curve is ‘real.’ With *Prunus*, this was true for the ‘s’ vulnerability curves constructed using the 27 and 42 cm diameter rotors. However, comparing the water extraction curve with the ‘r’-shaped 14 cm vulnerability curve for *Prunus*, the shift to cavitation water release occurred below *P*_50_. This indicates the early loss of conductance measured with the 14 cm diameter rotor is artefactual.

In comparing native versus vacuum degassed water extraction curves, we saw a significant shift in curve shape and the range of water potentials where the different phases of stored water were released. This was especially true for *Pinus*, *Fagus* and *Prunus*. Native water extraction curves were generally ‘s’ shaped, even for long-vesselled species and independent of rotor size, while vacuum degassed curves were not. Flushing might diminish the quality of water extraction curve measurements by introducing microbubbles, causing the effect and resulting in altered cavitation water release. Another possibility is that flushing, especially with the vacuum infiltration technique, fills portions of the stem that are normally not filled with water and causes the entire stem to behave differently in terms of water release dynamics, especially in the initial phase (Wang *et al*. 2015). To avoid these problems, extraction curve samples should not be flushed prior to measurement. Furthermore, it is recommended to test and quantify the occurrence of native embolism in samples for extraction or vulnerability curves using direct observations, such as tomography, in order to avoid any bias.

## Conclusions

Our study confirmed that contaminants associated with the microbubble effect play a role in artefacts associated with centrifuge-based vulnerability curves, with the solution in the centrifuge reservoirs or the perfusion solution used to measure flow acting as the source of microbubbles. If the microbubbles can travel by buoyancy or mass flow from one vessel end to the axis of rotation, the microbubble effect will occur even if a vessel has an end-wall but is open to one end. While the Cavitron technique is robust to measure xylem resistance to cavitation in tracheid-bearing and short-vesselled species, when the maximum vessel length exceeds the sample length or when vessels are cut open from one sample end to the axis of rotation, the microbubble effect may induce artefactual cavitation. This was demonstrated by the comparison of vulnerability curves and water extraction curves, especially for *Prunus*, which has an intermediate vessel length among our study species. Flushing altered the water release phases of water extraction curves, and is not recommended. While native water extraction curve were highly repeatable, $P_{50}^{\prime }$ was more resistant than *P*_50_, indicating that water extraction curves are not a viable alternative to vulnerability curves. We did not compare native versus flushed vulnerability curves because previous studies have shown flushed vulnerability curves to have more vulnerable *P*_50_ in angiosperms (Choat *et al*. 2010). Future water extraction curve tests may compare ring porous versus diffuse porous species, and test samples with the bark intact.

## Sources of Funding

This work was supported by the programme ‘Investments for the Future’ (ANR-10-EQPX-16, XYLOFOREST) from the French National Agency for Research. A.L.P. was supported by National Science Foundation Graduate Research Fellowship (United States) (DGE-1326120), National Science Foundation Graduate Research Opportunities Worldwide Fellowship (United States) and Chateaubriand Fellowship (honorary) (France).

## Contributions by the Authors

All authors contributed to writing and reviewing the manuscript.

## Conflict of Interest Statement

None declared.

## Supporting Information

The following additional information is available in the online version of this article –

**Table S1.** The P12 and P88 from vulnerability curves using three different rotor sizes for five study species.

Additional Information
