# Supplementary material for: Testing the ‘microbubble effect’ using the Cavitron technique to measure xylem water extraction curves
Source: AoB Plants. 2016 Feb 22;8:plw011. doi: 10.1093/aobpla/plw011 (PMC4804203; doi:10.1093/aobpla/plw011)
Supplement: Additional Information [file supp_8_plw011_index.html]

Testing the ‘microbubble effect’ using the Cavitron technique to measure xylem water extraction curves — Testing the ‘microbubble effect’ using the Cavitron technique to measure xylem water extraction curves — Additional Information 

# Testing the ‘microbubble effect’ using the Cavitron technique to measure xylem water extraction curves

## Additional Information

Additional Information

- Additional Information - Docx file
