# Supplementary material for: Testing the ‘microbubble effect’ using the Cavitron technique to measure xylem water extraction curves
Source: AoB Plants. 2016 Feb 22;8:plw011. doi: 10.1093/aobpla/plw011 (PMC4804203; doi:10.1093/aobpla/plw011)
Supplement: Additional Information [file supp_plw011_plw011supp.docx]

**Supporting Information**

**Table S1**. The water potential at which 12% and 88% of hydraulic conductivity is lost (*P*_12_ and *P*_88_, respectively) with replicates (n), determined from vulnerability curves using the 14 cm, 27 cm, and 42 cm diameter rotors for five study species. *P*_12_ and *P*_88_ measured in the 14 cm diameter rotor for *Pinus* and *Fagus* could not be determined as curves were not run to completion due to the maximum rotational velocity of the rotor.

|  |  | **Vulnerability curves** | | |
| --- | --- | --- | --- | --- |
| **Species** |  | **14 cm rotor** | **27 cm rotor** | **42 cm rotor** |
| *Pinus pinaster* | *P*_12_ | -- | -3.31 ± 0.03 (5) | -2.70 ± 0.30 (5) |
|  | *P*_88_ | -- | -4.09 ± 0.13 (5) | -4.31 ± 0.10 (5) |
|  |  |  |  |  |
| *Populus nigra* | *P*_12_ | -1.60 ± 0.05 (5) | -1.82 ± 0.09 (9) | -1.77 ± 0.12 (6) |
|  | *P*_88_ | -2.77 ± 0.18 (5) | -2.82 ± 0.06 (9) | -2.67 ± 0.10 (6) |
|  |  |  |  |  |
| *Fagus sylvatica* | *P*_12_ | -- | -3.51 ± 0.13 (6) | -3.50 ± 1.12 (5) |
|  | *P*_88_ | -- | -4.60 ± 0.08 (6) | -4.51 ± 0.08 (5) |
|  |  |  |  |  |
| *Prunus cerasifera* | *P*_12_ | -1.05 ± 0.44 (10) | -4.32 ± 0.31 (9) | -5.16 ± 0.09 (6) |
|  | *P*_88_ | -7.57 ± 0.40 (10) | -7.49 ± 0.31 (9) | -6.74 ± 0.06 (6) |
|  |  |  |  |  |
| *Eucalyptus sp.* | *P*_12_ | -0.41 ± 0.06 (6) | 0.09 ± 0.28 (4) | 0.04 ± 0.22 (6) |
|  | *P*_88_ | -1.79 ± 0.25 (6) | -4.23 ± 0.45 (4) | -2.72 ± 0.56 (6) |
